# The Role of Biomarkers, Metabolomics, and COVID-19 in Venous Thromboembolism—A Review of Literature

**DOI:** 10.3390/ijms241713411

**Published:** 2023-08-29

**Authors:** Vittoriano Della Corte, Renata Riolo, Stefania Scaglione, Rosaria Pecoraro, Antonino Tuttolomondo

**Affiliations:** Internal Medicine and Stroke Care Ward, Department of Health Promotion, Maternal and Infant Care, Internal Medicine and Medical Specialities (PROMISE) “G. D’Alessandro”, University of Palermo, Piazza delle Cliniche n.2, 90127 Palermo, Italy

**Keywords:** venous thromboembolism, deep vein thrombosis, DVT, D-Dimer, thrombin, cytokines, metabolomics, COVID-19

## Abstract

In recent years, the field of venous thromboembolism has undergone numerous innovations, starting from the recent discoveries on the role of biomarkers, passing through the role of metabolomics in expanding our knowledge on pathogenic mechanisms, which have opened up new therapeutic targets. A variety of studies have contributed to characterizing the metabolic phenotype that occurs in venous thromboembolism, identifying numerous pathways that are altered in this setting. Among these pathways are the metabolism of carnitine, tryptophan, purine, and fatty acids. Furthermore, new evidence has emerged with the recent COVID-19 pandemic. Hypercoagulability phenomena induced by this viral infection appear to be related to altered von Willebrand factor activity, alteration of the renin–angiotensin–aldosterone system, and dysregulation of both innate and adaptive immunity. This is the first literature review that brings together the most recent evidence regarding biomarkers, metabolomics, and COVID-19 in the field of venous thromboembolism, while also mentioning current therapeutic protocols.

## 1. Introduction

The last few years have seen the maturation of numerous discoveries in the field of venous thromboembolism. In particular, more and more evidence has been reported regarding biomarkers, the role of metabolomics, and that of COVID-19, not to mention the advent of direct oral anticoagulants, which have rapidly established themselves among the treatment options. Deep vein thrombosis (DVT) and pulmonary embolism (PE) represent the major diseases of venous thromboembolism (VTE) [1]. DVT usually involves the deep veins of the lower or upper limbs but can occur in other sites. Occlusion of the deep veins in a limb by a thrombus damages drainage of blood, thereby leading to pain and swelling distal to the obstruction [1]. Pulmonary embolism refers to a block of a pulmonary artery by a thrombus that has traveled from elsewhere in the body, through the bloodstream, and to the lungs. DVT in the legs—or less commonly, the arms—is by far the leading source of pulmonary embolism [1]. The incidence of thromboembolism increases with increasing age. Women are usually affected at a younger age [2]. Approximately two-thirds of VTE cases are associated with deep vein thrombosis and 80% are proximal [2]. Distal (below the knee) DVTs are more transient episodes, whereas proximal DVTs are related to chronic conditions [2,3]. Deep venous thrombosis is frequently secondary to heritable and acquired risk factors [4]. Heritable risk factors include abnormalities associated with hypercoagulability of the blood, the most common of which are factor V Leiden and the prothrombin G20210A gene mutations [4]. Acquired risk factors include advanced age, history of previous VTE, obesity, and active cancer, all of which limit mobility and may be associated with hypercoagulability [5,6]. Superimposed on this background risk, VTE often occurs in the presence of triggering factors, which increase the risk above the critical threshold. In 25–50% of first episodes of DVT, no trigger is identified [7,8].

Triggering factors such as surgery, trauma, and pregnancy or estrogen therapy lead to endothelial cell activation, stasis, and hypercoagulability, which are the components of the Virchow triad [9,10]. In 25–50% of first episodes of DVT, no predisposing factor is identified [11]. The main complications involve the extension of thrombosis and the recurrence of PE and DVT [11]. Long-term complications include post-thrombotic syndrome (PTS), which is characterized by chronic venous symptoms and/or signs secondary to DVT [11]. This is the most frequent chronic complication of DVT and occurs in 30–50% of patients within 2 years of proximal DVT [11]. A previous ipsilateral DVT, proximal location (ileo-femoral > popliteal), and stenosis of residual veins are the most significant risk factors for PTS [12]. According to the recent guidelines, we can use the Villalta score for the diagnosis and treatment of PTS (post-thrombotic syndrome) [11,12,13]. In the diagnosis of DVT, clinical signs and symptoms remain the bedrock of diagnostic strategy, even if unspecific and variable. This is the first literature review that brings together the most recent evidence regarding biomarkers, metabolomics, and COVID-19 in the field of venous thromboembolism, while also mentioning current therapeutic protocols.

## 2. COVID and Venous Thromboembolism

In addition to possible metabolic alterations, it is necessary to mention that infectious processes can also be triggers of thromboembolic episodes. In this regard, it is essential to note SARS-CoV2 infection. The Coronavirus disease of 2019 (COVID-19) is caused by the SARS-CoV2 coronavirus. Thromboembolic complications have been reported in COVID-19 patients from different groups [14,15]. Organs such as the lungs, spleen, lower limbs, and brain are affected by the hypercoagulability phenomena induced by viral infection. In severe cases of the disease, these complications are associated with a high risk of mortality. SARS-CoV2 uses its spike protein (S protein) to bind to the human angiotensin-converting enzyme 2 (ACE2) receptor [16]. ACE2 is not only expressed at the level of hair cells in the nasopharynx but is also found in blood vessels, the heart, the brain, and the kidney [17]; it is a molecule that also regulates the activity of the renin–angiotensin–aldosterone system (RAAS) [17]. As a result of infection with COVID-19, downregulation of ACE2 occurs; consequently, the action of the RAAS system is altered, with altered blood flow and increased hypercoagulability phenomena [17]. To this aspect, it should be added that the immune/inflammatory condition alone is a risk factor associated with increased blood clotting [18]. With angiotensin II impairment, as a result of ACE2 alteration, the oxidative stress process via the NADPH pathway is enhanced [19]. This is followed by progressive endothelial dysfunction and overexpression of LOX-1, COX-2, and VEGF in the endothelium [19]. Endothelial dysfunction is also associated with endothelial expression of many prothrombotic molecules and receptors including P-selectins and angiopoietin 2 and endothelin 1, which are specific activators of thrombotic phenomena [19]. From the study of patients who died from COVID-19, compared with H1N1 influenza, it was found that angiogenesis and alveolar capillary microthrombi in COVID-19 were up to nine times more prevalent than in flu [19]. This phenomenon appears to be related to altered von Willebrand factor (vWF) activity and dysregulation of both innate and adaptive immunity [19]. Since this is an infection that is a daily cause for scientific research, knowing that thrombotic processes are triggered on the one hand by dysregulation of RAAS and on the other by an excessive innate immune response to SARS-CoV2 may offer new opportunities for the development of innovative therapies for the treatment of COVID-19-induced coagulopathy.

## 3. Biomarkers in Venous Thromboembolism

**Figure 1:**
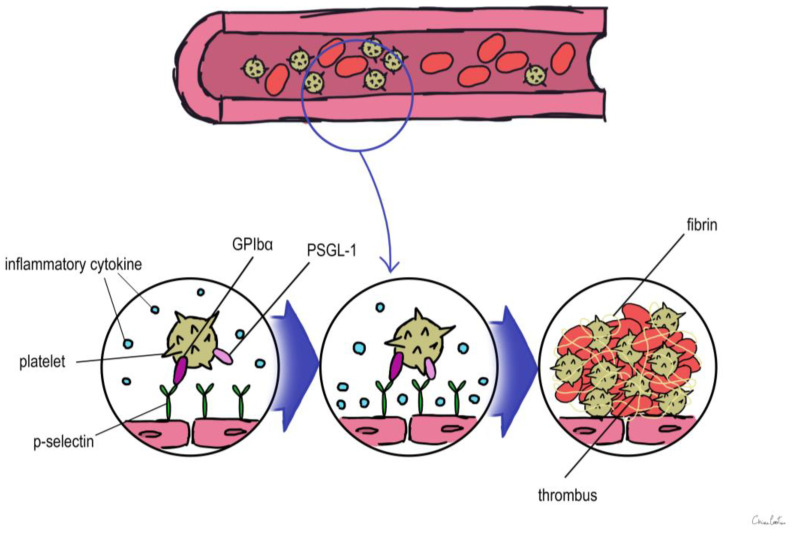
Overview of the role of biomarkers during thrombus formation. PSGL-1: P-selectin glycoprotein ligand 1.

Considering that VTE can often present with few symptoms, it would be useful to know biomarkers that enable early identification of patients at high or low risk of primary and recurrent VTE. Various established and novel biomarkers associated with VTE have been investigated with regard to their potential for predicting primary or recurrent VTE, for facilitating the diagnosis of VTE, and for optimizing the clinical management of VTE. Actually, these biomarkers can be divided into two categories from the pathobiology of DVT or thrombotic disease. One is coagulation markers, such as D-dimer, Thrombin, etc. while the other is inflammatory markers, including P-selectin, inflammatory cytokines, and microparticles (Figure 1).

### 3.1. D-Dimer

D-Dimer is a cross-linked fibrin degradation product that forms right after throm-bin-generated fibrin clots are broken down by plasmin and indicates a general stimulation of blood coagulation and fibrinolysis [20]. Testing for D-Dimer was investigated as a tool for the diagnosis of VTE and has been incorporated into diagnostic algorithms in the management of patients with suspected VTE since D-Dimer levels rise during a critical incident of VTE [20,21,22]. D-Dimer is the best-recognized biomarker for the first assessment of suspected VTE; a negative result of D-Dimer may confidently rule out both DVT and PE with a high sensitivity of up to 95% and a negative predictive value of almost 100% [23]. D-Dimer testing must be incorporated into thorough sequential diagnostic methodologies that involve clinical probability assessment and imaging tools because of its poor specificity for proving VTE [23]. D-Dimer was examined as a risk factor for the occurrence of a future first incident of VTE and was related with a three-fold higher risk in a population-based cohort analysis [23]. Additionally, in prospective cohort studies, D-Dimer levels are a well-researched biomarker for the estimation of the risk of VTE recurrence following the cessation of oral anticoagulant therapy [23]. In subjects with prior unprovoked VTE, Palareti et al. measured D-Dimer levels 1 month after discontinuing oral anticoagulation and found that normal levels (500 ng/mL) had a high negative predictive value for VTE recurrence [24]. In a different investigation, Eichinger et al. demonstrated that elevated D-Dimer levels were linked to an even greater risk of recurrent VTE, particularly in individuals with congenital thrombophilia, such as a factor V Leiden or prothrombin variant [25]. On the basis of the above evidence, the authors Eichinger et al. concluded that the de-termination of the duration of oral anticoagulation for secondary VTE prevention may be influenced by the measurement of D-Dimer, which has become a pillar in the diagnostic work-up of patients with suspected VTE and is essential in the identification of hyper-coagulable conditions [25].

### 3.2. Thrombin

Thrombin is crucial for the acceleration of the coagulation cascade because it activates platelets, Factor V, and FVIII and because it is an essential part of a positive feedback loop that causes the production of a significant amount of additional thrombin, the conversion of fibrinogen to fibrin, and ultimately the formation of clots. Some studies in the past have demonstrated that TG is one of the risk factors for VTE and can be used as a predictive marker to assess thrombosis [26,27]. Many authors across the years, such as Lutsey et al. [28] and Vilieg et al., have tried to show the lack of an association between increased TG level and the recurrence of VTE; however, some investigators thought TG parameters alone were inappropriate for the exclusion of DVT or to predict the risk of recurrence of VTE [29].

### 3.3. P-Selectin

P-selectin, which is stored in the granule membrane of resting platelets (a-granules) and endothelial cells (Weibel–Palade bodies) [30], is a member of the selectin family of cell adhesion molecules together with E-selectin and L-selectin [31]. The primary ligand for P-selectin in vivo is P-selectin glycoprotein ligand 1 (PSGL-1), which is expressed in the majority of leukocytes and is also present in trace levels on platelets [31]. Transmembrane P-selectin is redistributed onto the cell surface after cell activation and partially discharged into the bloodstream in its soluble form (sP-selectin) [31]. It facilitates the interaction of leukocytes that express PSGL-1 with activated platelets and endothelial cells [32,33,34]. In humans, elevated levels of soluble P-sel (sP-sel) are typical in DVT and VTE [34]. The interaction between P-selectin and PSGL-1 is crucial for thrombus development [35,36,37]. P-selectin was shown to have an impact on fibrin deposition in the thrombus by Palabrica et al. [36]. They discovered that inhibiting P-selectin interactions selectively prevented fibrin from being deposited on a thrombogenic graft in a baby as well as leukocyte adherence to platelets [36].

Korne l Miszti-Blasius et al. [37] discovered that PSGL-1-null mice had milder thrombocytopenia, less fibrin deposition, and a smaller number of thrombosed blood vessels after giving collagen with epinephrine to wild-type and PSGL-1 knockout mice. As a result, it is conceivable that a lack of PSGL-1 might prevent leukocyte–platelet interactions and lessen the likelihood of thrombus development [37]. Using sP-sel as a biomarker may improve the positive predictive value (as determined by a positive duplex ultrasound). According to a study evaluating the use of sP-sel in combination with a Wells risk prediction score for diagnosing VTE, this combination may be able to rule in the diagnosis of DVT with a sensitivity of 91% (low sP-sel and low Wells score to rule out the diagnosis) and a specificity of 98% (high sP-sel and high Wells score to rule in the diagnosis) [38].

In conclusion, a significant antithrombotic impact was seen when PSGL-1 and P-selectin interacted [38]. As a result, focusing on P-selectin or its ligand PSGL-1 may offer a viable treatment strategy for clinical circumstances. Numerous studies have revealed that the P-selectin–PSGL-1 interaction induces a procoagulant state by causing the formation of leukocyte-derived microparticles [39] and mediating the transfer of tissue factor (TF) to platelets [40]. This is in addition to its roles in mediating the binding of platelets and endothelial cells with leukocytes and enhancing fibrin deposition [40].

### 3.4. Inflammatory Cytokines

An increasing number of studies point to a role for inflammatory markers in VTE, including CRP and interleukin (IL)-1b, 6, 8, and 10. An initiator of the extrinsic route of coagulation, TF, may be affected by inflammatory cytokines, potentially setting off thrombotic illness [41]. Recent laboratory investigations have shown that elevated CRP levels significantly impact the development of VTE [42,43].

It is likely that mutations in genes encoding for proteins involved in inflammation may affect susceptibility to VTE based on the link between inflammation and coagulation [44]. Inflammation-related gene polymorphisms were looked at by Beckers et al. in both the VTE patient and control groups. It was discovered that IL-1A, IL-4, IL-6, and IL-13 Polymorphisms were linked to the development of VTE [45].

Several authors [46,47] have researched an association between proinflammatory cytokines and VTE. Reitsma and Rosendaal’s [46,47] case-control study not only showed an association with cytokines such as IL-1 beta, IL-6, IL-10, and TNF alpha but also revealed a deterioration of endothelial function in patients with VTE [46,47].

According to research by Downing et al. [48], exogenous IL-10 supplementation reduced inflammation and thrombus development whereas IL-10 neutralization enhanced thrombosis and inflammation. They suggested that IL-10 may be employed therapeutically to treat VTE [48].

### 3.5. MPs (Microparticles)

MPs, which are by definition between 0.1 and 1.0 lm in size, are small membranous vesicles released from the plasma membranes of platelets, leukocytes, red cells, and endothelial cells in response to apoptosis or cellular activation [49,50]. In the past, MPs were considered cellular debris without a biological function. In recent years, several studies have highlighted its role in inflammation and vascular function [51].

In the context of hypercoagulable conditions, elevated MP values have been found, and above all they have been highlighted in patients with VTE [52]. Moreover, MPs are the primary carriers of circulating TF, the principal initiator of intravascular thrombosis. However, at the moment, there are not enough studies (only animal studies) that clarify their role.

A new horizon in the management of VTE may be the application of metabolomics profiling in the area of vascular diseases, which can become a game changer in early diagnosis and patient management.

## 4. Metabolomics

The term “metabolomics” refers to the comprehensive profile of all low-molecular-weight compounds; intermediate- or end-products of metabolism; derived from the biochemical and physiological processes of the organism; and present in biological fluids, cells, and tissues [53]. Metabolites are the downstream expression of the transcriptome, genome, and proteome, reflecting the phenotype of an individual at the time of sample collection [53]. The term of these molecules can be influenced by genetics, disease, diet, lifestyle, and drug intake [54].

**Figure 2:**
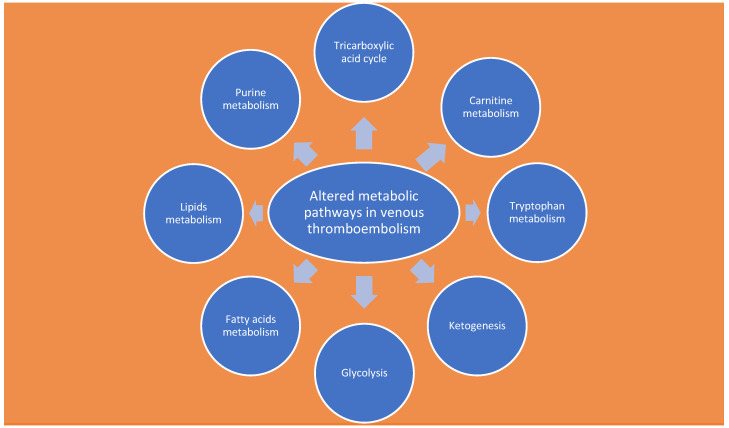
Altered metabolic pathways in venous thromboembolism.

Knowing metabolic phenotypes can help in understanding the molecular mechanisms underlying the development or progression of some diseases, seen as changes in genes and proteins due to disease (Figure 2) [55].

The field of metabolomics has been developed and utilized to identify potential biomarkers for early disease diagnosis [56,57]. However, there is currently a scarcity of studies conducted on human subjects. This method is highly sensitive, to the extent that even slight changes in sample collection or analysis can significantly influence the obtained results [56]. Metabolic phenotyping can be approached through targeted and untargeted methods [56,57]. The targeted approach involves the identification of specific metabolites that are characteristic of particular metabolic pathways [56,57]. In contrast, metabolite profiling aims to identify potential alterations in metabolites and signaling pathways [56,57]. The ultimate goal is to identify disease biomarkers and attainable therapeutic targets [54,56,57]. The analysis of metabolites, on the other hand, aims to identify potential alterations in metabolites and signaling pathways, with the goal of identifying disease biomarkers and achievable therapeutic targets [54].

### Analysis of Metabolites Involved

Maekawa et al. conducted the inherent metabolite study on animals, especially rabbits [58]. The study revealed that metabolites such as lactic acid, glycine, glutamate, cysteine glutathione disulfide, glutamine, and lysine were prominently present in venous thrombus [58]. The concentrations of these metabolites in the thrombus were found to be at least five times higher than in vein blood [58]. Fresh venous thrombus from the studied rabbits exhibited distinct levels of metabolites associated with glycolysis, purine metabolism, and tryptophan metabolism compared to vein blood [58]. In this regard, Chen et al. [59] showed that tryptophan would appear to have a protective role as it is associated with a reduction in PE mortality rate by 90 percent and also seems to reduce the incidence of thrombosis by 60 percent [59].

Voils S. A. et al. [60], in a study involving traumatized patients with and without venous thromboembolism, identified potential metabolites that can interact with significant proteins [60]. In this regard [60], the analysis of eight genes (AFMID, CCBL1, CCBL2, IDO1, IDO2, KMO, KYNU, TDO2) [60] involved in the tryptophan pathway metabolites, such as N-formylkynurenine or 5-hydroxy-N-formylkynurenine, revealed that three genes in particular (KYNU, CCBL1, and CCBL2) may have distinct concentrations in individuals developing VTE [60]. However, future studies are needed to validate these results in different populations using a multiomics approach [60].

Cristiana Bulato et al. [61], studying two prothrombin variants (p.Arg596Leu and p.Arg596Gln) associated with antithrombin resistance in VTE patients, identified a novel substitution affecting Arg596 of the prothrombin molecule in an Italian family known as Padua 2, which significantly increases the risk of PE [61].

Jiang et al. [1] hypothesized that both long-chain and short-chain carnitines may collaborate in the hemostatic system and pathophysiological features of VTE. Additionally, triacylglycerols, phosphatidylethanolamines, and amino acids seem to have an impact on the processes of DVT and PE (refer to Table 1) [1].

Obi et al. [55], on the other hand, conducted a metabolomic analysis of blood in mice with experimentally induced thrombosis, revealing higher levels of glutamine, phenylalanine, and proline in the blood of older animals compared to younger ones [55]. These three metabolites showed a correlation with venous wall impairment and levels of P-selectin in the venous wall [55]. The study suggests that increased concentrations of glutamine, phenylalanine, and proline, particularly in association with aging, is a possible consequence of decreased enzyme activity in their metabolism [55]. The altered activity of these metabolites appears to be related to the increased oxidative stress observed in aging individuals [66,67].

Other studies conducted on animals [68,69] have revealed that trimethylamine-*N*-oxide (TMAO), a metabolite of choline, can contribute to the aggravation of metabolic diseases by inducing epigenetic changes, increasing platelet reactivity, and promoting thrombosis formation [68,69].

The concentration of TMAO is believed to stimulate multiple platelet agonists [70], leading to platelet hyperresponsiveness, modulation of platelet function, and increased calcium release from intracellular stores [70].

Metabolomics studies have also been conducted for PE [62].

Bujak et al. [62], in their study on swine models of pulmonary embolism, identified alterations in metabolites involved in glycolysis, lipid production, and ketone body metabolism [62]. Specifically, they observed changes in metabolites related to glycolysis, lipid metabolism, and ketone bodies [62]. The findings indicate that altered carnitine and triglyceride concentrations can affect energy metabolism in DVT and potentially trigger episodes of acute PE [62,63].

Hiroshi Deguchi et al. [63], through a case-control study using liquid chromatography–mass spectrometry, observed reduced concentrations of acylcarnitines (ACs) in the plasma of VTE patients compared to matched controls [63]. Based on these findings, it was hypothesized that plasma levels of acylcarnitines (ACs) might be associated with the risk of VTE [63,64].

In the analysis of neoplastic patients who have an increased risk of VTE, Belghasem et al. [65] found that blood levels of kynurenine and hydroxyl sulfate (tryptophan metabolites) were elevated [65]. These metabolites act as ligands of the aryl hydrocarbon receptor (AHR) signaling pathway [65]. Plasma from xenograft-bearing mice activated the AHR pathway, resulting in increased levels of tissue factor (TF) and plasminogen activator inhibitor 1 (PAI-1) in venous endothelial cells in an AHR-dependent manner [65]. Pharmacological inhibition of AHR activity led to a reduction in TF and PAI-1 levels in endothelial cells, resulting in decreased thrombotic mechanisms [65].

Observational studies [71,72] have suggested that circulating metabolite concentrations and serum albumin levels are associated with an increased risk of VTE [71]. However, it remains unclear whether these observations establish a cause-and-effect relationship [71]. In cancer patients, for instance, a decrease in serum albumin is considered an indicator of overall health decline and poor prognosis [71], and this reduction is also associated with an elevated risk of thromboembolism [71]. Similar findings have been observed in patients with nephrotic syndrome, where the risk of pulmonary embolism increases proportionally with the decrease in serum albumin levels [40].

Liu et al. [73] conducted a study to evaluate the correlation between reduced serum albumin levels and thromboembolic risk and found an interesting association [73]. They documented that not only is there a correlation between these two factors but also that low levels of monounsaturated fatty acids (MUFA) and the ratio of MUFA to total fatty acids can increase the risk of VTE [73].

In another study by Morelli et al. [74], the correlation between lipid levels and the risk of pulmonary embolism was assessed. The literature review revealed that this correlation is influenced by factors such as sex, age, race, comorbidities, body mass index (BMI), statin use, and type 2 diabetes mellitus [74]. Lipid analysis showed an inverse association between apo B and apo A1 levels and venous thrombosis. Decreasing levels of both apolipoproteins increased the risk of venous thrombosis, while accounting for potential confounding factors [74]. Although apo B and apo A1 levels were associated with several hemostatic and CRP factors, none of these factors demonstrated a direct correlation with the risk of venous thrombosis [74]. Interestingly, a protective role of Apo B against VTE emerged from the analysis [74]. In contrast, there is strong evidence linking high levels of apo B and LDL-C with an increased risk of arterial disease [75]. Considering the different pathophysiology of venous and arterial thrombosis, the data analysis revealed that TC, LDL-C, HDL-C, and triglyceride levels do not appear to be associated with the risk of PE [74]. The study by Bergner et al. on chronic venous ulceration of the lower extremities [76], through biological liquids analysis, identified significant metabolic differences [76]. By serum analysis, L-carnitine was the most altered metabolite [76]. In fact, this metabolite was increased in the healed group compared with the unhealed group [76]. Increased carnitine in enrolled patients could support new cell generation and subsequent healing [55]. In addition, ceramides were found to be metabolites with altered concentrations. Ceramides consist of sphingosines, which are part of the fatty acid wall [77]. The literature review shows that ceramides are found to be of increased concentration in almost all stress processes including inflammation, heat, ultraviolet light, hypoxia, and oxidative stress [77]. Recent literature has also documented that ceramides play an essential role in cellular metabolism to the extent that they regulate both fatty acid activity and protein synthesis [78], and also control the action of the Akt pathway. This molecule represents a serine/threonine kinase that regulates signaling pathways of cell growth and subcellular distribution of nutrient transporters. In addition, Akt activates anabolic pathways while attenuating catabolic ones [79]. In light of the findings in the literature, it is possible that the reduced levels of ceramide in the group of healed patients may activate Akt and allow the ulcer to heal [79]. This aspect is even more supported by the literature, as activation of the Akt/mTOR signaling pathway contributes to wound healing [80].

## 5. Treatment of Venous Thromboembolism

Since 1899, the inherent topic of venous thromboembolism (VTE) prevention and the importance of hydration to maintain adequate circulation, elastic compression, and the need for patient movement, have been addressed [81]. Since 1937, heparin has been an effective drug for preventing thrombosis after cardiac surgery and trauma [82] and has emerged as a cornerstone drug in treating venous thromboembolism [83]. However, over time, through extensive research, a more systematic approach to VTE has been suggested [84]. Drug choice to date is strictly individualized and related to the patient’s thromboembolic and hemorrhagic risk [84].

VTE should be treated in the following cases [84]:

- Proximity deep vein thrombosis (DVT) of the lower extremity;
- Symptomatic distal DVT (calf vein);
- Symptomatic upper extremity DVT (axillary-subclavian veins);
- Pulmonary embolism (PE);
- Subsegmental EP in a patient at risk of recurrence;
- Surveillance for subsegmental EP in a patient without proximal DVT and a reduced risk of recurrence.

Following the diagnosis of EP, it is necessary to immediately perform drug treatment [85,86].

Current treatment options include the use of the following [87,88,89]:

- Low molecular weight heparin;
- Fondaparinux;
- Unfractionated heparin;
- Oral anticoagulants directed against factor Xa or thrombin inhibitors.

Today, we talk about personalized therapy, as the goal is to attend to patient’s clinical characteristics and comorbidities [88,89]. Pharmacological approaches are different and consider pregnant women, neoplastic patients, or patients with severe renal failure or creatinine clearance <30 mL/min [88,89].

Low-molecular-weight heparin finds greater use in patients with active malignancy or in pregnant women with an impaired renal function such that direct oral anticoagulants (DOACs) cannot be used [90].

DOACs are drugs that have found wide use in recent years due to their efficacy and safety, as demonstrated by the Aristotle studies for apixaban [91], Rocket for rivaroxaban [92], RE-LY for dabigatran [93], and ENGAGE AF-TIMI for edoxaban [94].

Based on new evidence [84], for patients with acute VTE, factor Xa inhibitors (apixaban, edoxaban, rivaroxaban) and direct thrombin inhibitors (dabigatran) have become first-line oral anticoagulants for long-term treatment in most patients [84]. Furthermore, DOACs, unlike warfarin, turn out to be more manageable drugs, do not require routine monitoring of INR values, and have no particular food interactions [95]. In this regard, James C. Coons et al. [95] showed that the effectiveness of these drugs is also found in obese patients with venous thromboembolism. Obese patients with a BMI over 40 treated with DOAC did not show a statistically significant difference compared to patients treated with warfarin in relapses of EP and DVT and did not experience any considerable bleeding [95].

Going into detail, the treatment of DVT has three stages (Table 2) [11]:

- The initial phase (about 5–21 days after diagnosis): patients, depending on clinical features, receive either treatment initially parenterally and then switch to treatment with vitamin K antagonists (VKAs), or begin treatment with high-dose therapy [11]
- Long-term treatment: therapy with VKA or DOAC 3–6 months after diagnosis [11].

With regard to DOACs, this translates into the following dosages: apixaban 10 mg bid for the first seven days, then 5 mg bid or 2.5 mg bid if the patient’s bleeding risk is considered; rivaroxaban 15 mg bid (30 mg/day) for the first twenty-one days, then 20 mg/day or after consideration of bleeding risk; edoxaban 60 mg/day or 30 mg/day with regard to actual functionality by low-molecular-weight heparin for 5–10 days; dabigatran 150 mg bid preceded by low-molecular-weight heparin for the first 5–10 days [11].

Regarding the role of DOACs in neoplastic patients, one cannot fail to mention the HOKUSAI CANCER VTE study [96]. This study documented the non-inferiority of edoxaban to dalteparin in the treatment of VTE in neoplastic patients [96]. Regarding the possibility of apixaban use, mention should be made of the Caravaggio study [97], which demonstrated the non-inferiority of apixaban to dalteparin without increased risk of significant bleeding [96]. However, this study had limitations considering that patients with neoplasms with a high risk of bleeding were not found to be enrollable [97].

- Prolonged treatment (after the initial 3–6 months): The decision to prolong treatment (beyond the first 3–6 months) is related to the benefit/risk ratio of continuing anticoagulant therapy and must be tailored on every single patient [97].

In patients with severe renal impairment (creatinine clearance <30 mL/min), a high risk of bleeding, or impaired renal activity, unfractionated heparin (UFH) administered intravenously might be preferred. There are few studies about the possible efficacy of unfractionated heparin in obese (BMI > 40 kg/m2) and underweight (<50 kg) patients [97].

Finally, in patients for whom anticoagulant therapy is not feasible, possible alternative therapeutic strategies are as follows [97]:

- Thrombosis, usually employed in patients with acute thromboembolism and hemodynamic instability;
- Vena cava filter in patients for whom anticoagulant therapy is absolutely contraindicated [97];
- Elastic compression and rapid mobilization have also been found to be effective in the treatment of DVT—however, careful caution is needed in patients with severe peripheral arterial disease [97].

## 6. Conclusions

From the literature [98], it emerges that the most highly expressed metabolites in TEP included lipids, branched-chain amino acids (BCAAs), glutamate, taurine, lactate, and myoinositol identified in venous tissue. Overexpressed metabolites in venous leg ulcers, on the other hand, included lactate, BCAAs, lysine, 3-hydroxybutyrate, and glutamate identified in wound fluid and ulcer biopsies [98].

VTE cases were associated with reduced levels of carnitine, overregulated aromatic amino acids, 3-hydroxybutyrate, BCAAs, and lipids in the plasma, serum, thrombus, and vein wall; dysfunction of the kynurenine and tricarboxylic acid pathways have been reported [98].

Future research should focus on studies aimed at analyzing the metabolites involved in order to identify either useful markers of early diagnosis of TEP or potential molecular targets of personalized drug therapies. 

## Figures and Tables

**Table 1:** Specific metabolite category changes in the context of DVT or PE [55,56,57,58,62,63,64,65].

Metabolite	Disease	Directionality and Context of the Changes
Hypoxanthine	DVT	Raised in jugular vein thrombus, compared to venous blood levels (in rabbit)
Serotonin	DVT	Raised in jugular vein thrombus, compared to venous blood levels (in rabbit)
Guanine	DVT	Raised in jugular vein thrombus, compared to venous blood levels (in rabbit)
Taurine	DVT	Raised in jugular vein thrombus, compared to venous blood levels (in rabbit)
AMP	DVT	Raised in jugular vein thrombus, compared to venous blood levels (in rabbit)
3-Hydroxykynurenine	DVT	Raised in jugular vein thrombus, compared to venous blood levels (in rabbit)
Lactic acid	DVT	Raised in jugular vein thrombus, compared to venous blood levels (in rabbit)
Citric acid	DVT	Reduced in jugular vein thrombus, compared to venous blood levels (in rabbit)
Glucose 6-phosphate	DVT	Reduced in jugular vein thrombus, compared to venous blood levels (in rabbit)
NADP	DVT	Reduced in jugular vein thrombus, compared to venous blood levels (in rabbit)
Tryptophan	DVT	Reduced in jugular vein thrombus, compared to venous blood levels (in rabbit)
Methionine sulfoxide	DVT	Reduced in jugular vein thrombus, compared to venous blood levels (in rabbit)
Cysteine glutathione disulfide	DVT	Reduced in jugular vein thrombus, compared to venous blood levels (in rabbit)
Glutamine	DVT	Raised in whole blood samples of old mice with venous thrombosis (VT) of inferior vena cava, compared with young mice with VT and age-matched controls without VT
Phenylalanine	DVT	Raised in whole blood samples of old mice with VT of inferior vena cava, compared with young mice with VT and age-matched controls without VT
Proline	DVT	Raised in whole blood samples of old mice with VT of inferior vena cava, compared with young mice with VT and age-matched controls without VT
Glycerol	PE	Raised in venous blood samples of pigs with PE compared to pigs without PE.
Pyruvic acid	PE	Raised in venous blood samples of pigs with PE compared to pigs without PE.
Lactic acid	PE	Raised in venous blood samples of pigs with PE compared to pigs without PE.
Palmitic acid	PE	Raised in venous blood samples of pigs with PE compared to pigs without PE.
Oleic acid	PE	Raised in venous blood samples of pigs with PE compared to pigs without PE.
3-hydroxybutyric acid	PE	Raised in venous blood samples of pigs with PE compared to pigs without PE.
10:1 Acylcarnitines	PE	Reduced in venous blood samples of patients who had PE 3 months before compared with patients without history of PE.
16:1 Acylcarnitines	PE	Reduced in venous blood samples of patients who had PE 3 months before compared with patients without history of PE.

DVT—Deep Vein Thrombosis; PE—Pulmonary Embolism; VT—Venous Thrombosis.

**Table 2:** Therapeutic protocols in DVT [11].

Initial Phase VTE	Dosing	5–21 Days after Diagnosis
APIXABAN	10 mg bid	for the first 7 days
RIVAROXABAN	15 mg bid	for the first 21 days
EDOXABAN	60 mg/day or 30 mg/day with regard to actual functionality by low-molecular-weight heparin	for 5–10 days
DABIGATRAN	150 mg bid preceded by low-molecular-weight heparin	for the first 5–10 days
**Long-Term Treatment**	**Dosing**	**Days after Initial Phase**
APIXABAN	5 mg bid/2.5 mg bid	for 3–6 months
RIVAROXABAN	20 mg die	for 3–6 months
EDOXABAN	60 mg/day or 30 mg/day with regard to actual functionality	for 3–6 months
DABIGATRAN	150 mg bid ore 110 mg die	for 3–6 months
VKA	Dosing related INR value	for 3–6 months
LOW-MOLECULAR-WEIGHT HEPARIN	Dosing is typically weight-based and continued at the same dose used for initial anticoagulation.	for 3–6 months

## Data Availability

No new data were created or analyzed in this study. Data sharing is not applicable to this article.
